# Laminarin from Irish Brown Seaweeds *Ascophyllum nodosum* and *Laminaria hyperborea*: Ultrasound Assisted Extraction, Characterization and Bioactivity

**DOI:** 10.3390/md13074270

**Published:** 2015-07-10

**Authors:** Shekhar U. Kadam, Colm P. O’Donnell, Dilip K. Rai, Mohammad B. Hossain, Catherine M. Burgess, Des Walsh, Brijesh K. Tiwari

**Affiliations:** 1 School of Biosystems and Food Engineering, University College Dublin, Belfield, Dublin 4, Ireland; E-Mails: shekhar.kadam@ucdconnect.ie (S.U.K.); colm.odonnell@ucd.ie (C.P.O.); 2 Food Biosciences, Teagasc Food Research Centre, Dublin 15, Ireland; E-Mails: dilip.rai@teagasc.ie (D.K.R.); mohammad.hossain@teagasc.ie (M.B.H.); 3 Food Safety, Teagasc Food Research Centre, Dublin 15, Ireland; E-Mails: Kaye.Burgess@teagasc.ie (C.M.B.); des.walsh@teagasc.ie (D.W.)

**Keywords:** laminarin, antioxidant, antimicrobial, bioactive, *Laminaria hyperborea*, *Ascophyllum nodosum*

## Abstract

Ultrasound assisted extraction (UAE), purification, characterization and antioxidant activity of laminarin from Irish brown seaweeds *Ascophyllum nodosum* and *Laminarina hyperborea* were investigated. UAE was carried out using 60% ultrasonic power amplitude and 0.1 M hydrochloric acid for 15 min. Separately, solid-liquid extraction was carried in an orbital shaker using 0.1 M hydrochloric acid at 70 °C for 2.5 h. UAE with hydrochloric acid resulted in the highest concentration of laminarin, 5.82% and 6.24% on dry weight basis from *A. nodosum* and *L. hyperborea*, respectively. Purification of all extracts was carried out using molecular weight cut off dialysis at 10 kDa. Characterization of the laminarin fraction was carried out using matrix assisted laser desorption/ionization time-of-flight mass spectrometry. Antioxidant activity of *A. nodosum* and *L. hyperborea* extracts had 2,2-diphenyl-1-picrylhydrazyl (DPPH) inhibition levels of 93.23% and 87.57%, respectively. Moreover, these extracts have shown inihibition of bacterial growth of *Staphylcoccus aureus*, *Listeria monocytogenes*, *Escherichia coli* and *Salmonella typhimurium.*

## 1. Introduction

Half of all global biodiversity is present in the marine ecosystem. Marine organisms are an excellent source of bioactive compounds. Marine algae, one of the important components of the marine ecosystem, are a good source of carotenoids, vitamins, minerals, phenolic compounds, sulphated polysaccharides, peptides and proteins [1]. These compounds have been demonstrated to possess diverse biological activities including anti-tumor, anti-viral, anti-coagulant, anti-allergic, anti-viral, anti-tumor and antibacterial [2]. Extracts from these seaweeds have been investigated for use as ingredients in food and feed products [3]. *Ascophyllum nodosum* is a brown seaweed which belongs to the Phaeophyceae class and it is the only species in the genus *Ascophyllum*. It is good source of fucoidan, alginates, ascophyllan, laminarins and polyphenols [4]. *Laminaria hyperborea* is a large, leathery brown seaweed from the Laminariaceae species which is found on the coast of Norway, Scotland and Ireland. It is commercially exploited by the hydrocolloid industry for alginate production [5]. Crude extracts from this seaweed are rich in phenolic compounds and β-glucan polysaccharides known as laminarins.

Laminarins are composed of (1,3)-β-d-glucan [6]. They consist of (1,3)-β-d-glucopyranose residues with some 6-*O*-branching in the main chain and some β-(1,6)-intrachain links are also present. The molecular weight of laminarin is approximately 5 kDa [7]. Both water soluble and insoluble forms of laminarin exist depending mainly on the level of branching [8]. In general, extraction of laminarin involves grinding, precipitation in an mild acid medium, ultrafiltration and dialysis [9]. Characterization of laminarin rich extracts can be carried out by high performance size exclusion chromatography using a refractive index detector [10], matrix assisted laser desorption/ionization time-of-flight (MALDI-TOF) with dehydroxy-benzoic acid as matrix [11] and nuclear magnetic resonance spectroscopy using the ^1^H-^13^C heteronuclear single quantum coherence method [12].

However, current methods of extraction of laminarin are time and energy intensive, and result in poor yields. The development of novel eco-friendly extraction techniques is required to improve extraction rate and yield. Ultrasound is a low cost, easy to use, environment friendly technology suitable for the extraction of bioactive compounds from natural sources [13]. There are no reported studies on the use of ultrasound assisted extraction for laminarin applications. Only a limited number of studies on the characterization of laminarin-rich extract from brown seaweeds are available in the literature [6,11,12]. This study investigates the efficiency of ultrasound assisted extraction of laminarin in comparison to traditional extraction methods, and characterizes the resultant laminarin rich extracts with respect to molecular weight, chemical properties in addition to antioxidant and antimicrobial activities.

## 2. Results and Discussion

### 2.1. Laminarin and Phenolics Content

In this study two seaweed species harvested from the west coast of Ireland were selected for the extraction of laminarin. Laminarin was extracted using ultrasound assisted extraction and conventional solid liquid extraction using water and 0.1 M HCl (Table 1).

**Table 1:** Laminarin and total phenolic content of different extracts.

Seaweed Species	Solvent Type	Extraction Method	Time (min)	Sample Code	Laminarin Assay (% db)	Total Phenolic Content (mg PGE/g_db_)
*Laminaria hyperborea*	Water	Ultrasound	15	LHWU	5.975 ± 0.467	0.365 ± 0.039
*Laminaria hyperborea*	0.1 M HCl	Ultrasound	15	LHAU	6.240 ± 0.008	0.343 ± 0.003
*Laminaria hyperborea*	Water	Solid liquid	150	LHWS	4.362 ± 0.197	0.363 ± 0.057
*Laminaria hyperborea*	0.1 M HCl	Solid liquid	150	LHAS	3.254 ± 0.235	0.352 ± 0.021
*Ascophyllum nodosum*	Water	Ultrasound	15	ANWU	5.290 ± 0.480	0.156 ± 0.014
*Ascophyllum nodosum*	0.1 M HCl	Ultrasound	15	ANAU	5.822 ± 0.343	0.128 ± 0.008
*Ascophyllum nodosum*	Water	Solid liquid	150	ANWS	4.599 ± 0.030	0.166 ± 0.015
*Ascophyllum nodosum*	0.1 M HCl	Solid liquid	150	ANAS	4.304 ± 0.165	0.110 ± 0.007

The highest laminarin content was measured in the extract of *L. hyperborea* and *A. nodosum* obtained using ultrasound and 0.1 M HCl. In previous studies, 0.1 M HCl was also found to give higher extraction yields compared to water [14]. Moreover HCl at higher temperatures is more effective than at room temperature for laminarin extraction [7]. Laminarin content varies with species, with *L. hyperborea* having a higher level of laminarin compared to *A. nodosum*. Laminarin content also varies with factors such as harvesting season and geographical location. Laminarin is absent during the period of fast growth in spring, but in autumn and winter, it may represent up to 35% of the dried weight of the fronds [15]. Ultrasound was found to give higher extraction yields of laminarin. High power ultrasound treatment for 15 min achieved a higher extraction yield than conventional solid and liquid extraction for both seaweeds investigated. Laminarin yield for *L. hyperborea* extracted using ultrasound was 36.97% and 91.76% higher using water and 0.1 M HCl solvents respectively, whereas for *A. nodosum* laminarin yield was 15.02% and 35.62% higher using water and 0.1 M HCl solvents respectively. This can be attributed to the bubble cavitation phenomena generated by ultrasound waves. The implosion of cavitation bubbles generates macroturbulence, high-velocity interparticle collisions, and perturbations in microporous particles of the biomass. Cavitation near liquid-solid interfaces directs a fast-moving stream of liquid through the cavity at the surface. Impingement by these microjets results in surface peeling, erosion, and particle breakdown, facilitating the release of bioactive compounds and other components from the biological matrix. These effects increase the efficiency of extraction by increasing mass transfer by eddy and internal diffusion mechanisms [16]. Ultrasound assisted extraction (UAE) has also been successfully used for extraction of *A. nodosum* bioactive compounds including phenolic compounds, fucose and uronic acids [17,18]. The phenolic content was higher in *L. hyperborea* extracts. Water was demonstrated to be a better solvent than HCl for extraction of phenolics. This may be attributed to fact that acid solvents at a temperature of 70 °C may be detrimental to phenolic compounds leading to a lower content in acid extracts. The highest content of phenolics observed in *L. hyperborea* and *A. nodosum* was 0.365 mg PGE/g_db_ and 0.166 mg PGE/g_db_, respectively.

### 2.2. Characterization of Extracts

**Figure 1:**
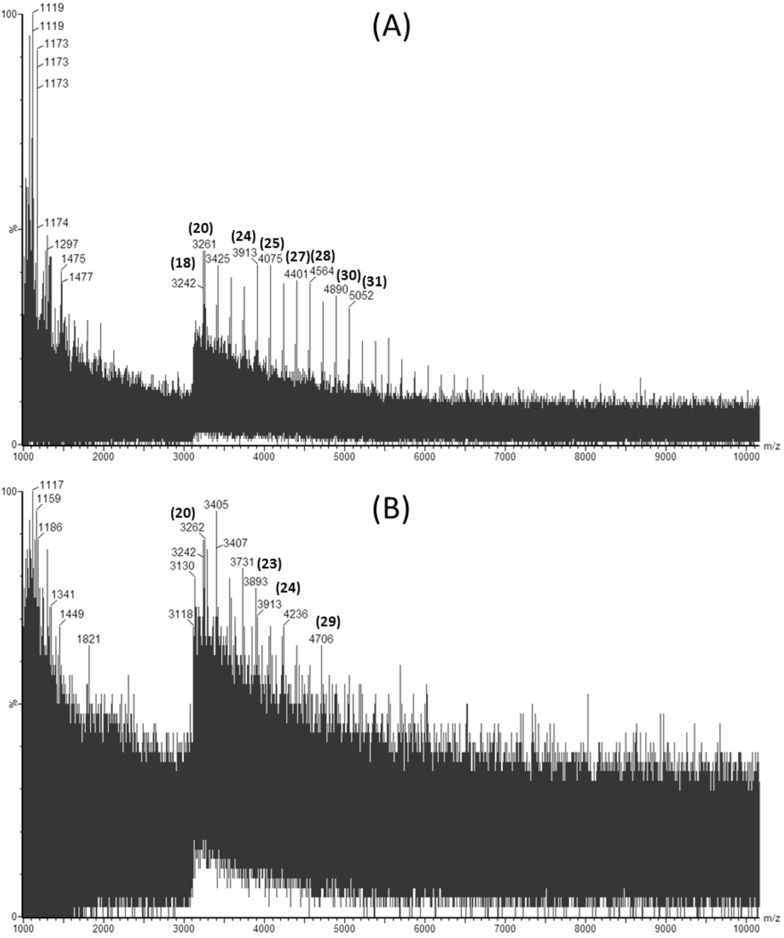
Matrix Assisted Laser Desorption Ionization Quadrupole Time-of-Flight Mass Spectrometry (MALDI-Q-TOF-MS) spectra showing the distribution of molecular weights of (**A**) commercial laminarin in sinapinic acid and (**B**) *Laminaria hyperborea* extract obtained by ultrasound assisted extraction using 0.1 M HCl as a solvent.

Laminarin rich extracts were analyzed for their molecular weight distribution using Matrix Assisted Laser Desorption Ionization Quadrupole Time-of-Flight Mass Spectrometry (MALDI-Q-TOF-MS). Figure 1A,B show the mass spectra obtained in negative ion mode for a laminarin standard and an ultrasound assisted extract of *L. hyperborea*, respectively.

The degree of polymerization (DP) for each laminarin peak is shown in bracketed bold numbers above the corresponding *m*/*z* peaks. Four extracts were evaluated to investigate the effect of ultrasound and seaweed species on extraction of laminarin. Since M-chains and G-chains in the native laminarins differ by only 2 mass units, they cannot be distinguished from high molecular weight profiles [11]. Laminarin extracted using UAE from *L. hyperborea* had higher molecular weight laminarins ranging from 3242 to 5052 Da (corresponding to DP20 to DP31) compared to the other extracts measured. Meanwhile, *L. hyperborea* obtained with conventional extraction yielded laminarins from DP20 to DP24 only. This demonstrates that ultrasound assisted extraction is more efficient than solid liquid extraction for extracting higher molecular weight laminarins. This can be attributed to the bubble cavitation phenomena releasing high molecular weight laminarins from *L. hyperborea*. Similar results were reported for extraction of high molecular weight phlorotannins from *A. nodosum* using ultrasound assisted extraction [18].

Ultrasound treated *A. nodosum* extracts yielded laminarins from DP25 to DP30 (molecular weights in the range of 4075 to 4884 Da). *A. nodosum* extracts did not yield lower molecular weight laminarins compared to *L. hyperborea* extracts. Moreover, *L hyperborea* extracts had a wide range of degree of polymerization compared to *A. nodosum* extracts.

### 2.3. Bioactivities of Laminarin Rich Extract

Laminarin has been found to possess various biological activities. The antioxidant and antimicrobial activities of the crude laminarin extract are shown in Table 2. The % of 2,2-diphenyl-1-picrylhydrazyl (DPPH) inhibition of seaweed extracts was found to be highest in ultrasound treated extracts using acid solvent for *L. hyperborea* (87.58%) and *A. nodosum* (93.24%). O’Sullivan *et al.* [19] also found that *A. nodosum* was one of the most effective extracts for scavenging DPPH radicals. The extracts obtained with acid were found to have higher antioxidant activity than those obtained using water solvent. Further, authors reported that *A. nodosum* with 0.45 g/100 g (Gallic acid equivalent) of phenolic content exhibited antioxidant activity of 25.6% DPPH inhibition [19]. Balboa *et al.* [20] have extensively reviewed antioxidant activities in brown seaweeds and they have reported that phenolic compounds possess antioxidant properties.

**Table 2:** Antioxidant activity as %DPPH inhibition and antimicrobial activity as minimum inhibition concentration (MIC) of different extracts.

Sample	Antioxidant Activity (% DPPH Inhibition)	MIC (mg/mL)			
		*E. coli*	*S. typhimurium*	*S. aureus*	*L. monocytogenes*
LHWU	52.78	21.0	21.0	5.3	2.6
LHAU	87.58	13.1	13.1	6.6	3.3
LHWS	73.91	45.6	22.8	11.4	5.7
LHAS	86.04	28.2	28.2	7.0	7.0
ANWU	61.46	43.2	21.6	NI ^*^	NI
ANAU	93.24	596.8	14.9	29.8	59.7
ANWS	15.13	NI	25.5	NI	NI
ANAS	87.82	NI	33.4	66.8	66.8

NI, no inhibition.

The extracts were tested for inhibition against two Gram positive (*Staphylcoccus aureus* and *Listeria monocytogenes*) and two Gram negative (*Escherichia coli* and *Salmonella typhimurium*) bacterial strains. All extracts of *L. hyperborea* were found to inhibit the growth of all the micro-organisms tested. The acid extracts had better inhibition of bacterial growth compared to water extracts. However, *A. nodosum* extracts only inhibited the growth of *S. typhimurium* completely. Only the acid extract of *A. nodosum* proved effective in inhibiting the bacterial growth of all species. The higher phenolic content and antioxidant activity of *L. hyperborea* extracts may enhance the antimicrobial efficacy of these extracts as many phenolic compounds have been shown to possess antimicrobial properties [21]. Laminarin rich extracts prepared using ultrasound and acid solvents had minimum inhibitory concentrations (MIC) of 13.1 mg/mL for *E. coli* and *S. typhimurium* and 6.6 mg/mL and 3.3 mg/mL for *S. aureus* and *L. monocytogenes*, respectively. This is the first reported study demonstrating that laminarin rich extracts possess microbial inhibitory activity. There are number of reports of seaweed extracts such as *Sargassum polyophyllum*, *Sargassum flavellum*, *Padina australis* and *Sargassum binderi* possessing antimicrobial activity [22]. However, ultrasound can be a novel alternative to high energy consuming traditional solid liquid extraction methods. These seaweed extracts have potential application in the preparation of antimicrobial products for example, a hydrogel wound dressing incorporating a seaweed *Polysiphonia lanosa* extract [23].

## 3. Experimental Section

### 3.1. Chemicals and Reagents

Laminarin produced from *Laminaria digitata*, 2,2-diphenyl-1-picrylhydrazyl (DPPH), phloroglucinol, sodium carbonate, Folin-Ciocalteu reagent (FCR), and hydrochloric acid (37%) were obtained from Sigma-Aldrich (Wicklow, Ireland). GOPOD (glucose oxidase/peroxidase) reagent and β-glucosidase were obtained from Megazyme, Wicklow, Ireland.

### 3.2. Seaweed Samples

Brown seaweed *A. nodosum* and *L. hyperborea* were harvested from Finavarra, Co. Clare, Ireland in May 2014. Seaweed samples were washed thoroughly with fresh water to remove epiphytes and salt. Fresh seaweed samples were freeze dried. Dried seaweed was powdered using a hammer mill. Samples were stored at 4 °C prior to extraction studies.

### 3.3. Ultrasound Assisted Extraction

Ten grams of *A. nodosum* and *L. hyperborea* powders were extracted using 200 mL of solvent (distilled water or 0.03 M HCl). HCl was used as the solvent for extraction based on preliminary studies and previously reported studies for the extraction of laminarin [24,25,26]. A 750 W ultrasonic processor (VC 750, Sonics and Materials Inc., Newtown, CT, USA) with a 13 mm diameter probe and constant frequency of 20 kHz was used. Ultrasonic energy was controlled by setting the amplitude of the sonicator probe. Ultrasound treatment was applied for 15 min at an amplitude level of 60% which corresponds to an ultrasonic intensity of 35.61 W cm^−2^. Ultrasonic power dissipated was calculated at each amplitude level, with temperature (*T*) recorded as a function of time (*t*) under adiabatic conditions using a T-type thermocouple. From temperature *versus* time data, the initial temperature rise *dT/dt* was determined by polynomial curve fitting. The ultrasonic power (*P*) was determined using Equation (1) where *dT/dt* is the change in temperature over time (°C s^−1^), *C_p_* is the specific heat of water (4.18 kJ kg^−1^ °C^−1^), and *m* is the mass (kg).

(1) $$P=mC_{p}{\left(dT/dt\right)}_{t=0}$$

Ultrasonic intensity (W cm^−2^) dissipated from an ultrasonic probe tip with diameter *D* (cm) is given by Equation (2)

(2) $$UI=\frac{4P}{\pi D^{2}}$$

The traditional solid-liquid method of extraction involved stirring at 70 °C for 2.5 h using distilled water and 0.1 M HCl as solvents and no ultrasound pretreatment was employed. The extracted samples were then centrifuged at 9000 rpm for 30 min. The supernatant was separated and precipitated with ethanol overnight at 4 °C. The precipitated extract was freeze dried and stored at −20 °C for further analysis. The different methods of extraction carried out in this experiment are listed in Table 3. The extraction yield (%) was calculated by measuring the mass of freeze dried extract over the initial mass of the sample.

**Table 3:** Different methods of extraction and corresponding sample codes.

Seaweed Species	Solvent Type	Extraction	Time (min)	Sample Code
*Laminaria hyperborea*	Water	Ultrasound	15	LHWU
*Laminaria hyperborea*	0.1 M HCl	Ultrasound	15	LHAU
*Laminaria hyperborea*	Water	Solid liquid	150	LHWS
*Laminaria hyperborea*	0.1 M HCl	Solid liquid	150	LHAS
*Ascophyllum nodosum*	Water	Ultrasound	15	ANWU
*Ascophyllum nodosum*	0.1 M HCl	Ultrasound	15	ANAU
*Ascophyllum nodosum*	Water	Solid liquid	150	ANWS
*Ascophyllum nodosum*	0.1 M HCl	Solid liquid	150	ANAS

### 3.4. Laminarin Assay

Laminarin in the extract was quantified by measuring the glucose concentration released by the enzymatic hydrolysis of laminarin [27]. A 100 μL sample volume was incubated in 100 μL of β-glucosidase enzyme at 40 °C for 15 min. After incubation, 3 mL of GOPOD (glucose oxidase/peroxidase) reagent was added. This mixture was incubated at 40 °C for 20 min. Finally the absorbance of the sample was measured at 510 nm by UV-VIS spectrophotometer (UV3100PC, VWR International). Laminarin produced from *Laminaria digitata* was used as a standard.

### 3.5. Total Phenolic Content

Total phenolic content was determined using the method of Wang *et al.* [28]. Folin-Ciocalteau reagent was diluted with distilled water at a ratio of 1:10. An extract of 100 μL was mixed with 100 μL of diluted Folin-Ciocalteau reagent and 100 μL of sodium bicarbonate (20%, w/v) was added to the mixture and diluted to 1000 μL with distilled water. This solution was maintained at room temperature for 30 min and the absorbance was measured at 735 nm by UV-VIS spectrophotometer (UV3100PC, VWR International). Results were expressed as mg phloroglucinol equivalents (PGE)/g_db_.

### 3.6. Matrix Assisted Laser Desorption Ionization Quadrupole Time-of-Flight Mass Spectrometry (MALDI-Q-TOF-MS)

Mass spectrometry of samples was performed using MALDI-Q-TOF-MS Waters Corporation, Milford, MA, USA). Prior to analysis, samples were dialyzed (molecular weight cut off of 10 kDa) in distilled water overnight. Aliquots of 5 μL of sample were mixed with 5 μL of matrix sinapinic acid. Finally 1–2 μL of sample was plated on a 96 well stainless steel MALDI plate. Samples were allowed to dry and co-crystallize with the matrix at room temperature and the plate was loaded in MALDI-Q-TOF mass spectrometer. Mass spectral data were obtained in the negative-ion mode for a mass range of *m/z* 1000 to *m/z* 10,000.

### 3.7. Antioxidant Activity—DPPH Method

DPPH free radical scavenging inhibition assay was used to determine the antioxidant capacity of extracted samples [29]. Extract sample of 200 μL was added to 800 μL of 60 μM DPPH in ethanol, decrease in absorbance was monitored at 517 nm by UV-VIS spectrophotometer (UV3100PC, VWR International) after 30 min incubation in dark. The readings were compared with the controls, which contained 200 μL of water instead of the seaweed extract. The percent inhibition was calculated as

(3) $$\text{\% Inhibition}=\frac{{\text{Abs}}_{\text{control}}-{\text{Abs}}_{\text{sample}}}{{\text{Abs}}_{\text{control}}}\times 100$$

### 3.8. Antimicrobial Activity—Iodonitrotetrazolium Chloride (INT) Dye Method

#### 3.8.1. Bacterial Strains and Culture Conditions

Seaweed extracts were tested for antimicrobial activity against the following strains of bacteria: *Staphylcoccus aureus* NCTC 8178, *Escherichia coli* DSM 1103, *Listeria monocytogenes* NCTC 11994 and *Salmonella typhimurium* SARB 65. The strains were stored on ceramic beads in glycerol at −80 °C prior to use. A bead of each strain was streaked on a nutrient agar plate and incubated for 18 h at 37 °C. A single colony was removed from each plate and inoculated into tubes containing 25 mL of sterile Mueller-Hinton Broth (MHB) and incubated for 22 h at 37 °C. Overnight cultures were vortexed and aliquots diluted appropriately in sterile MHB to produce solutions containing log_10_ 6.0 ± 0.5 cells/mL. Cell numbers were confirmed by plate counting. Antibiotic Gentamicin (0.2 mg/mL) was used as standard for negative growth.

#### 3.8.2. Minimum Inhibitory Concentration (MIC) Assay

The MIC of each seaweed extract was carried out using a previously described microtitre method of Kenny, Smyth, Walsh, Kelleher, Hewage and Brunton [21]. Each extract (2 mg/mL) was prepared by dissolving the material in distilled water added to the first well of the plate followed by a serial dilution across the plate. Iodonitrotetrazolium chloride (INT) dye was used identify microbial growth [30]. The MIC of each extract against a bacterial strain was determined as the lowest sample concentration at which no pink color appeared. This process was repeated in triplicate for each bacterial strain to ensure reproducibility.

## 4. Conclusions

In this study, water and acid extracts from *L. hyperborea* and *A. nodosum* were obtained using ultrasound assisted extraction and solid liquid extraction. The extracts were purified to obtain laminarin rich extracts. The *L. hyperborea* extracts contained higher contents of laminarin. The overall laminarin content in all the extracts was low which may be attributed to seasonal and geographical factors. Ultrasound was demonstrated to be a more efficient method of extraction than solid liquid extraction based on laminarin content and molecular weight distribution observed in the extracts. The laminarin rich extracts were also studied for biological activities including anti-oxidant and anti-microbial activity. This study is the first report of laminarin rich extracts possessing anti-microbial activity. The use of laminarin as a nutraceutical ingredient should be further investigated due to its dietary fiber properties in addition to the anti-oxidant and anti-microbial activities reported in this paper.
